# Antibody response to BNT162b2 mRNA vaccine in healthcare workers and residents in a long‐term care facility

**DOI:** 10.1111/ggi.14342

**Published:** 2022-01-10

**Authors:** Takao Kitagawa, Yasuhiro Kuramitsu, Kouji Nakagawa, Tohru Ohta, Kozo Akino, Masahiro Asaka, Masanobu Kobayashi

**Affiliations:** ^1^ Advanced Research Promotion Center Health Sciences University of Hokkaido Ishikari‐Tobetsu Japan

Dear Editor,

Starting on 12 April 2021, Japan began administering coronavirus disease 2019 vaccines to senior citizens aged ≥65 years. As of September 2021, >80% of them already completed two doses of vaccination. As a result, the new cases of infections among older adults have decreased and the incident of clusters in long‐term care facilities (LTCFs) have dramatically reduced. Coronavirus disease 2019 vaccine booster shots are considered in Japan at this point due to the waning immunity. To determine who should receive the booster shot, it is necessary to investigate the antibody response by the different age group and situation.

We compared the serological response induced by two doses of the mRNA vaccine, BNT162b2, between LTCF residents and healthcare workers (HCWs).

Written informed consent was obtained from all participants. If residents lacked the capacity to consent, the responsible guardian's permission was obtained. This study was approved by Health Sciences University of Hokkaido Research Ethics Boards (20N037045). A total of 75 residents of a LTCF and 69 HCWs of the LTCF and the affiliated hospital were included in the present study. Those previously infected with SARS‐Cov‐2 were excluded from this study. Almost all residents had more than two comorbidities and required assistance for activities of daily living. Four groups were compared: group 1: HCWs aged ≤64 years; group 2: HCWs aged between 65 and 84 years; group 3: LTCF residents aged between 65 and 84 years; and group 4: LTCF residents aged ≥85 years. Blood samples were collected at 28–45 days after the second injection using the BNT162b2 mRNA vaccine.

The quantitative enzyme‐linked immunosorbent assay test to measure an anti‐SARS‐Cov‐2 immunoglobulin G antibody to S1 protein was carried out by using the Vitros Immunodiagnostic Product anti‐SARS‐Cov‐2 S1 Quant immunoglobulin G test (Ortho Clinical Diagnostics, Raritan, NJ, USA) according to the manufacturer's instructions. Results were expressed as binding activity units per mL (BAU/mL; positive threshold: 17.8 BAU/mL; upper limit: 4000 BAU/mL).

For comparative analysis of antibody levels between four groups, the Kruskal–Wallis test followed by the Mann–Whitney *U*‐test, using the Bonferroni correction with adjustment of the probability (*P* < 0.05 / 4 = 0.0125) was carried out.

After vaccination, all residents tested positive for antibodies, except two residents. However, the median antibody titers were eightfold and fivefold lower in group 3 (median antibody titer 127 BAU/mL) and group 4 (median antibody titer: 200 BAU/mL), respectively, compared with those of group 1 (median antibody titer 1095 BAU/mL; *P* = 7.25465E‐08 and 1.11602E‐14, respectively; Fig. 1). Compared with the median antibody titers of group 1, those of group 2 tended to be lower, but the difference was not significant (*P* = 0.26341; Fig. 1). However, the median antibody titers of group 2 were higher than those of group 3 (*P* = 0.000415; Fig. 1).

**Figure 1:**
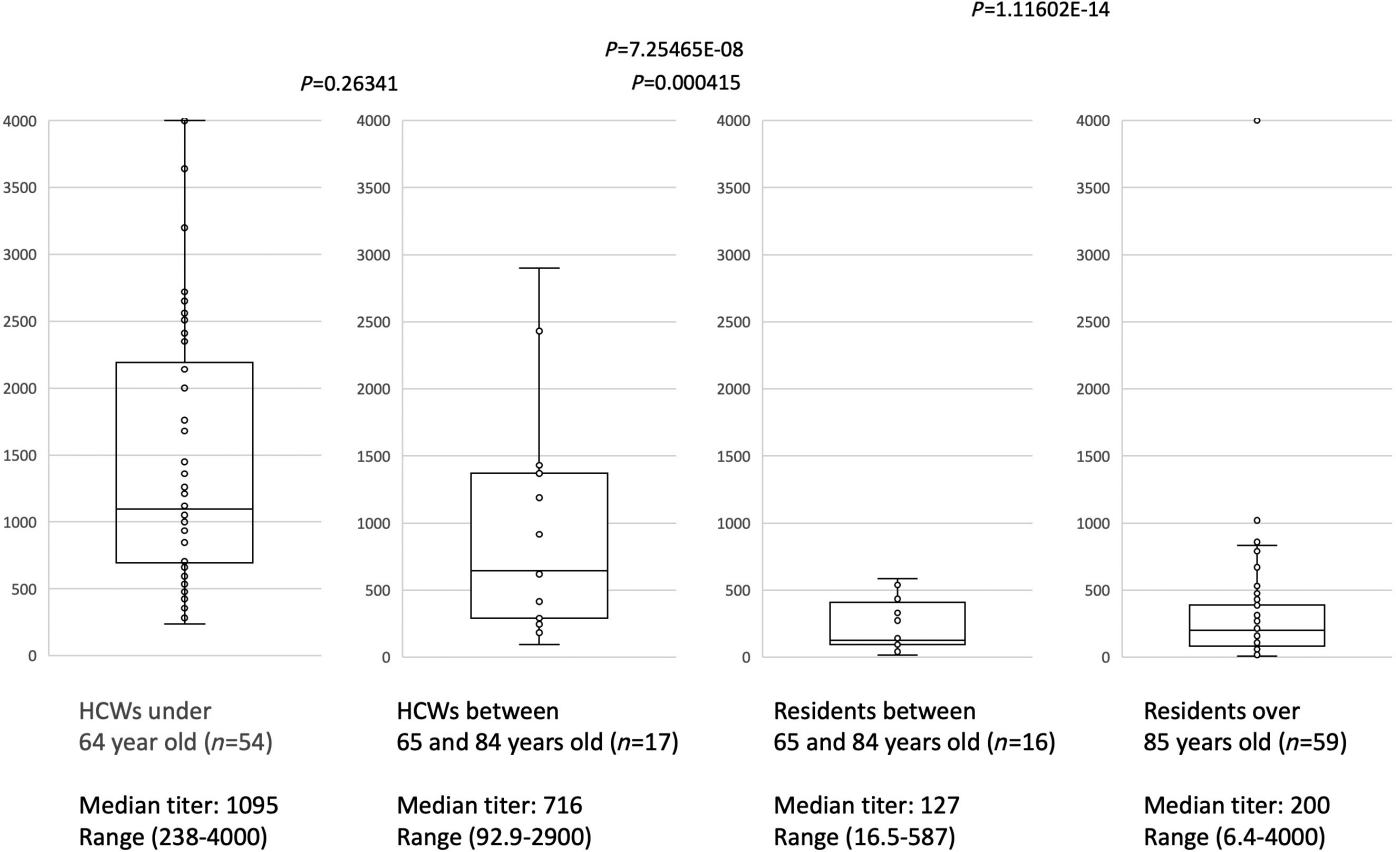
Serological response induced by two doses of the mRNA vaccine, BNT162b2. The quantitative SARS‐Cov‐2 spike antibody titers (expressed as BAU/mL) in 75 long‐term care facility residents and 69 healthcare workers (HCWs) 28–45 days after the second injection of BNT162b2 mRNA vaccine are shown. Medians with interquartile ranges are shown.

The present study found that serological response to two doses of the mRNA vaccine, BNT162b2, in LTCF residents was significantly lower than those in HCWs aged between 65 and 84 years, as well as in HCWs aged <64 years. This finding is in accordance with previous studies that compared the response to the vaccination in LTCFs and HCWs.^1^, ^2^ In addition, the present study showed that the serological response of older HCWs did not show any significant difference compared with that of younger HCWs, in accordance with previous studies that examined the response to the vaccination among older adults.^3^, ^4^ These results suggest that LTCF residents are thought to be immunocompromised by a variety of factors in addition to aging, such as comorbidity, malnutrition and inactivity,^5^ and that LTCF residents should be included in the target population for the third booster vaccine. Limitations include the small sample size, possible selection bias, failure to identify factors other than aging and failure to investigate cellular immunity.

## Disclosure statement

The authors declare no conflict of interest.

## Data Availability

The data that support the findings of this study are available from the corresponding author upon reasonable request.
